# Stereotactic radiosurgery for pilocytic astrocytoma: A single center retrospective study

**DOI:** 10.1007/s10143-026-04209-w

**Published:** 2026-03-21

**Authors:** Sukran Senyurek, Ilayda Kayir, Dogu Cihan Yildirim, Ali Haluk Duzkalir, Mehmet Orbay Askeroglu, Selcuk Peker

**Affiliations:** 1 Department of Radiation Oncology, Koç University Hospital, Istanbul, Türkiye; 2 Bahçeşehir University School of Medicine, Istanbul, Türkiye; 3 Department of Neurosurgery, Gamma Knife Center, Koç University Hospital, Istanbul, Türkiye; 4 Department of Health Physics, Institute of Health Sciences, Istanbul Medipol University, Istanbul, Türkiye

**Keywords:** Pilocytic astrocytoma, Gamma Knife radiosurgery, Progression-free survival

## Abstract

**Introduction:**

Pilocytic astrocytoma (PA) is a WHO grade I glioma with generally favorable outcomes; however, deep or eloquent tumor locations often limit safe gross total resection. Gamma Knife radiosurgery (GKRS) has emerged as a minimally invasive alternative for residual or recurrent disease.

**Materials and methods:**

A retrospective review of 88 patients (60 diagnosed in childhood and 28 in adulthood) treated with GKRS between 2008 and 2024 was conducted. Clinical, radiological, and treatment parameters were analyzed. PFS was assessed with Kaplan–Meier analysis, and predictive factors were identified using univariate and multivariate Cox regression models.

**Results:**

Median follow-up was 36 months. Pediatric patients showed 5- and 10-year PFS rates of 84.7% and 66.7%, while adults demonstrated 81.7% at both time points. Multivariate analysis identified prior radiotherapy as the only independent predictor of recurrence in the entire cohort, with significant impact also observed in the pediatric subgroup. Cystic/mixed morphology increased recurrence risk in pediatric patients, while no independent predictors were identified in adults.

**Discussion and conclusion:**

GKRS provides durable long-term tumor control in both pediatric and adult PA patients. Prior radiotherapy consistently predicts poorer PFS, underscoring its importance in treatment planning. In pediatric patients, tumor morphology also influences outcomes. Despite the retrospective design and cohort heterogeneity, this represents one of the largest single-center GKRS series and supports GKRS as a safe and effective modality for PA management. Prospective studies are needed to refine patient selection and optimize therapeutic strategies.

## Introduction

Pilocytic astrocytomas are rare, typically non-infiltrative astrocytoma and generally considered low-grade, slow-growing, well-circumscribed and surgically curable brain tumors [1]. These features cause that pilocytic astrocytomas have favorable prognosis and potential for curative treatment through gross total resection (GTR). These neoplasms are classified as World Health Organization (WHO) Grade I gliomas and are commonly present in the pediatric population and young adults [2].

Surgical resection remains the cornerstone of management, with GTR being the most significant predictor of recurrence-free survival, both in pediatric and adult populations. Despite their typically benign behavior, the location of these tumors—often near midline structures or within sensitive areas such as the brainstem or optic pathways—can pose challenges for complete surgical excision. When resection is incomplete, adjuvant treatments are required. Fractionated radiotherapy is effective but carries risks of long-term toxicity, making it a second-line option for unresectable recurrences. Chemotherapy is not first-line but has shown efficacy in selected contexts [3]. These limitations have prompted interest in stereotactic radiosurgery (SRS), particularly Gamma Knife radiosurgery (GKRS), as a focused, minimally invasive treatment for recurrent pilocytic astrocytomas (PAs).

Adult series suggest that GKRS provides durable local tumor control and favorable overall survival, although progression-free survival may be influenced by delayed cyst formation and tumor location [4–7]. In pediatric populations, GKRS has demonstrated excellent outcomes, with reported 5-year overall survival rates exceeding 95% and progression-free survival rates above 70% [8, 9]. Optimal results are typically observed in patients with small tumor volumes and no prior radiotherapy exposure. However, concerns regarding long-term neurocognitive and developmental effects persist, and most studies are limited by small sample sizes and heterogeneous follow-up.

In this study, we retrospectively reviewed patients with pilocytic astrocytoma treated with Gamma Knife radiosurgery. By evaluating outcomes across age groups, this study aims to clarify treatment safety and efficacy and support clinical decision-making in PAs management.

## Methods

### Study design and patient selection

We retrospectively reviewed patients with pilocytic astrocytoma treated with Gamma Knife radiosurgery (GKRS) from 2008 to 2024. Inclusion criteria were histologically confirmed pilocytic astrocytoma, treatment with GKRS, and adequate clinical and imaging follow-up. Patients with insufficient follow-up were excluded for survival analysis.

Patients were classified as pediatric, or adult based on age at diagnosis (< 18 vs. ≥ 18 years). All primary analyses (Kaplan–Meier and Cox regression) were performed according to this diagnosis-age stratification. Because pilocytic astrocytoma may be diagnosed in childhood but treated with GKRS later, we also report the distribution of age at GKRS descriptively; however, this variable was not used to define the pediatric/adult subgroups for survival analyses.

### Clinical and radiological evaluation

Baseline data included age, sex, tumor size and location, prior surgery, presenting symptoms, prior radiotherapy, and chemotherapy. Follow-up MRI was generally performed every three months during the first year after GKRS. In patients with radiologically stable disease, the imaging interval was gradually extended to every six months and subsequently to annual evaluations at the discretion of the treating physician.

Radiological response assessment was performed according to institutional criteria consistent with RANO definitions for low-grade gliomas [10]. A complete response (CR) was defined as the complete disappearance of both enhancing and non-enhancing tumor components. Partial response (PR) was defined as a ≥ 50% reduction in tumor volume, whereas stable disease (SD) was defined as a change of less than 25% in tumor volume. Progressive disease (PD) was defined as a ≥ 25% increase in the volume of either enhancing or non-enhancing tumor components. Radiation-related imaging changes were interpreted in conjunction with clinical findings and serial imaging follow-up to differentiate true progression from transient post-treatment effects.

### Radiosurgical technique

All patients were treated with single-fraction Gamma Knife stereotactic radiosurgery (GKRS) using the Leksell Gamma Knife^®^ 4 C, Perfexion, or Icon™ systems (Elekta Instrument AB, Stockholm, Sweden). Stereotactic MRI was used for target delineation. Treatment planning was performed jointly by a neurosurgeon and a radiation oncologist.

The prescribed marginal dose was determined individually based on tumor size, location, proximity to critical structures, and prior treatment history. Although a history of prior radiotherapy was taken into consideration during treatment planning, no fixed dose-reduction protocol was applied. Dose selection was individualized to optimize tumor control while respecting normal tissue constraints. Target delineation was performed on contrast-enhanced stereotactic MRI. The gross tumor volume (GTV) was defined as the contrast-enhancing lesion. No additional margin was applied; therefore, the planning target volume (PTV) was equivalent to the GTV.

### Statistical analysis

The primary outcome of the study was progression-free survival (PFS), and the secondary outcome was the identification of predictive factors associated with recurrence. PFS was defined as the time from Gamma Knife radiosurgery (GKRS) to either radiographic or clinical progression. Survival curves were estimated using the Kaplan–Meier method and compared using the log-rank test.

All 88 patients were consecutively treated during the study period. Four patients did not have post-treatment clinical and radiological follow-up data available at our institution and were therefore excluded from survival analyses, as progression-free survival could not be reliably determined.

To identify factors associated with recurrence, univariate analyses were performed using the log-rank test for categorical variables and Cox proportional hazards regression for continuous variables. Variables with a p-value ≤ 0.20 in univariate analysis were subsequently entered into a multivariate Cox proportional hazards regression model to determine independent predictors of recurrence. Hazard ratios (HRs) with 95% confidence intervals (CIs) were reported. The proportional hazards assumption was assessed graphically using log-minus-log survival plots.

## Results

### Patient characteristics

Among the 88 consecutive patients treated with GKRS during the study period, 60 were diagnosed before 18 years of age and 28 at 18 years or older. Based on the age at the time of Gamma Knife radiosurgery (GKRS), 50 patients received treatment during childhood and 38 during adulthood.

Patients were classified into pediatric (< 18 years) and adult (≥ 18 years) groups based on age at diagnosis. All subgroup analyses, including survival analyses, were performed according to this classification. Patient characteristics by diagnostic age are presented in Table 1.

The median age at GKRS was 15 years (range, 3–55), with a median of 11 years (3–29) in the pediatric group and 28 years (20–55) in the adult group. Across the cohort, 56 patients (63.6%) had undergone subtotal resection (STR), 21 (23.9%) gross total resection (GTR), and 11 (12.5%) biopsies. GKRS was performed as a salvage treatment in 70% of cases.

**Table 1:** Baseline characteristics according to age at diagnosis

Median Age at GKRS (range)	All Patients (*N* = 88)	Pediatric (*N* = 60)	Adult (*N* = 28)
	15 (3–55)	11 (3–29)	28 (20–55)
Gender			
Male	38	31	7
Female	50	29	21
Neurological Deficit Before GKRS			
No	59	39	20
Visual problems	7	6	1
Hearing problems	1	1	0
Ataxia	5	5	0
Other	16	9	7
First Surgery			
GTR	21 (23.9%)	18	3
STR	56 (63.6%)	38	18
Biopsy	11 (12.5%)	4	7
Second Surgery			
No	68	43	25
Yes	20	17	3
Tumor Location			
Cerebellum	33 (37.5%)	25	8
Lobar	19 (21.6%)	11	8
Brainstem	20 (22.7%)	11	9
Ventricular	9 (10.2%)	7	2
Other	7 (8.0%)	6	1
Tumor Type			
Solid	43 (48.9%)	28	15
Cystic	14 (15.9%)	8	6
Mix	31 (35.2%)	24	7
Prior Chemotherapy			
No	85	58	27
Yes	3	2	1
Prior Radiation Therapy			
No	79	53	26
Yes	9	7	2
Intent the GKRS			
First treatment	11 (12.5%)	4	7
Adjuvant treatment	15 (17%)	8	7
Salvage Treatment	62 (70.5%)	48	14
Recurrence After GKRS			
No	71	48	23
Yes	13	9	4
NA	4	3	1

*Abbreviations*: *N* Number of patients, *GKRS* Gamma Knife radiosurgery, *GTR* Gross total resection, *STR* Subtotal resection

### Details of GKRS

GKRS was delivered in a single fraction in all cohort. The median treatment volume was 2.7 cm³ (range, 1–9.3), the median prescribed dose was 12 Gy (8–15), the median prescription isodose line was 50% (40–60), and the median maximum dose was 24 Gy (16–44). Treatment parameters by age group are summarized in Table 2.

**Table 2:** Details of treatment parameters

Treatment Parameters	All (*N* = 88)	Pediatric (*N* = 60)	Adult (*N* = 28)
Treatment volume (cm3) (median, range)	2.7 (0.7–9.3)	3.6 (1-9.3)	2 (0.7-9)
Prescribed Dose (Gray), (median, range)	12 (8–15)	12 (8–15)	12 (10–15)
Prescription isodose line (%) (median, range)	50 (40–60)	50 (40–50)	50 (40–60)
Max dose (Gray) (median, range)	24 (16–44)	24 (16–44)	24 (20–33)
Treatment time (median, range)	30 (9–82)	31 (9–82)	27 (12–64)

### Survival Outcomes

**Fig. 1:**
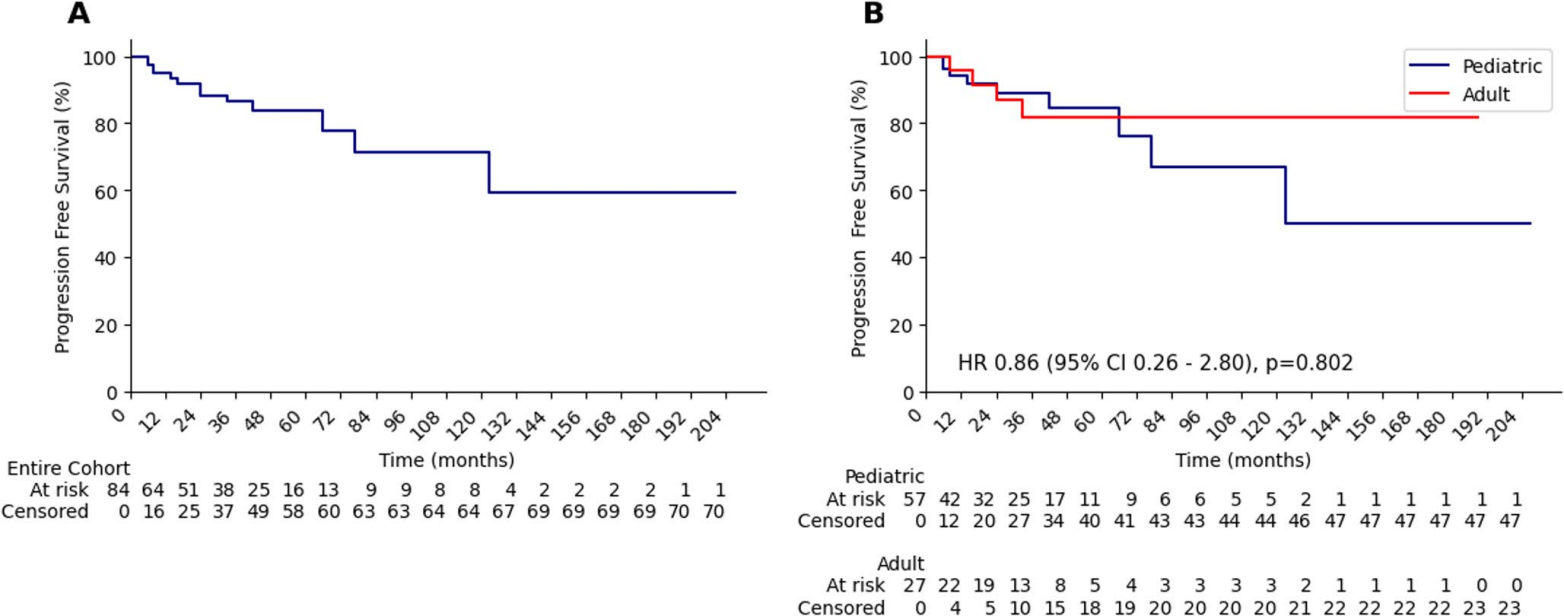
Kaplan–Meier progression-free survival (PFS) curves following Gamma Knife radiosurgery. (**A**) In the overall cohort, (**B**) Pediatric and adult subgroups

Among the 88 treated patients, recurrence patterns were evaluated in 84 patients with available follow-up data, median follow-up was 36 (6-207) months. Of these, 57 were pediatric and 27 were adults. Following GKRS, the cumulative incidence of recurrence was 15.5%, with recurrence observed in 5.2% of patients at 1 year and 11.6% at 2 years. In the pediatric cohort, the 1-year and 2-year recurrence rates were 5.8% and 10.8%, respectively, whereas in the adult cohort, the recurrence rates were 4.0% and 13.1% (P = 0.802). All patients were alive at the time of the last follow-up. In the overall cohort, the median progression-free survival (PFS) was not reached; the 5-year and 10-year PFS rates were 83.6% and 71.2%, respectively (Figure 1A). In the pediatric subgroup, the 5- and 10-year PFS rates were 84.7% and 66.7%, whereas in the adult subgroup, they were 81.7% and 81.7%, respectively (Figure 1B).

Univariate regression analysis was performed to identify predictors of recurrence following GKRS, including age, neurological deficit, treatment volume, initial surgical approach, indication for GKRS, tumor type, tumor location, and history of prior radiotherapy (RT) or chemotherapy (CT) in the entire cohort as well as in the pediatric and adult subgroups. Detailed results of the univariate and multivariate analyses are presented in Table 3.

**Table 3:** Univariate and Multivariate Analysis Outcomes

Parameters		All			Pediatric			Adult	
	Uni- *p* value	Multi-*p* value	Multi-HR (95% CI)	Uni-*p* value	Multi-*p* value	Multi-HR (95% CI)	Uni-*p* value	Multi-*p* value	Multi-HR (95% CI)
Age at diagnosis Continuous	0.61	-	-	0.43	-	-	**0.058**	0.36	1.04 (0.94–1.16)
Deficit Before GKRS Yes vs. No	**0.02**	0.06	3.4 (0.9–12.7)	**0.19**	0.057	5.3 (0.94–30.2)	**0.06**	0.36	3.76 (0.21–66.02)
Treatment volume Continuous	0.53	-	-	0.48	-	-	0.70	-	-
First surgery type GTR/STR vs. Bx/No	0.99	-	-	0.61	-	-	0.37	-	-
Tumor type Solid vs. Cystic/Mix	0.51	-	-	**0.15**	**0.01**	13.1 (1.6-102.9)	0.46	-	-
Tumor location Brainstem vs. Other	**0.23**	0.58	1.49 (0.35–6.2)	0.95	-	-	**0.12**	0.69	1.76 (0.1–30.7)
Prior RT Yes vs. No	**0.006**	**0.012**	6.2 (1.5–26.2)	**0.006**	**0.003**	19.5 (2.6-136.6)	0.74	-	-
Time to GKRS Continuous	0.50	-	-	0.42	-	-	0.93	-	-
Prior CT Yes vs. No	**0.12**	0.55	0.55 (0.07–3.9)	**0.10**	0.733	1.3 (0.2–9.5)	0.76	-	-
Treatment Intent Adj. vs. Salvage.vs Pri.	0.80	-	-	0.68	-	-	0.61	-	-

*Abbreviations*: Uni-p: Univariate analysis P value, *Multi-p* Multivariate analysis p value, *Multi-HR* Multivariate analysis Hazards ratio, *CI* Confidence Interval, *GKRS* Gamma Knife radiosurgery, *GTR* Gross total resection, *STR* Subtotal resection, *Bx* Biopsy, *RT* Radiation therapy, *CT* Chemotherapy, *Adj* Adjuvant, Pri Primer

In the entire cohort, pre-GKRS neurological deficit and prior RT were identified as significant factors in the univariate analysis, whereas tumor location and prior CT showed borderline significance. All of these variables were included in the multivariate model. Multivariate analysis demonstrated that a history of prior RT was the only independent predictor of recurrence after GKRS. In the pediatric subgroup, multivariate analysis revealed that cystic/mixed tumor morphology and prior RT were associated with an increased risk of recurrence. In contrast, no variable reached statistical significance as a predictor of recurrence in the adult subgroup.

**Fig. 2:**
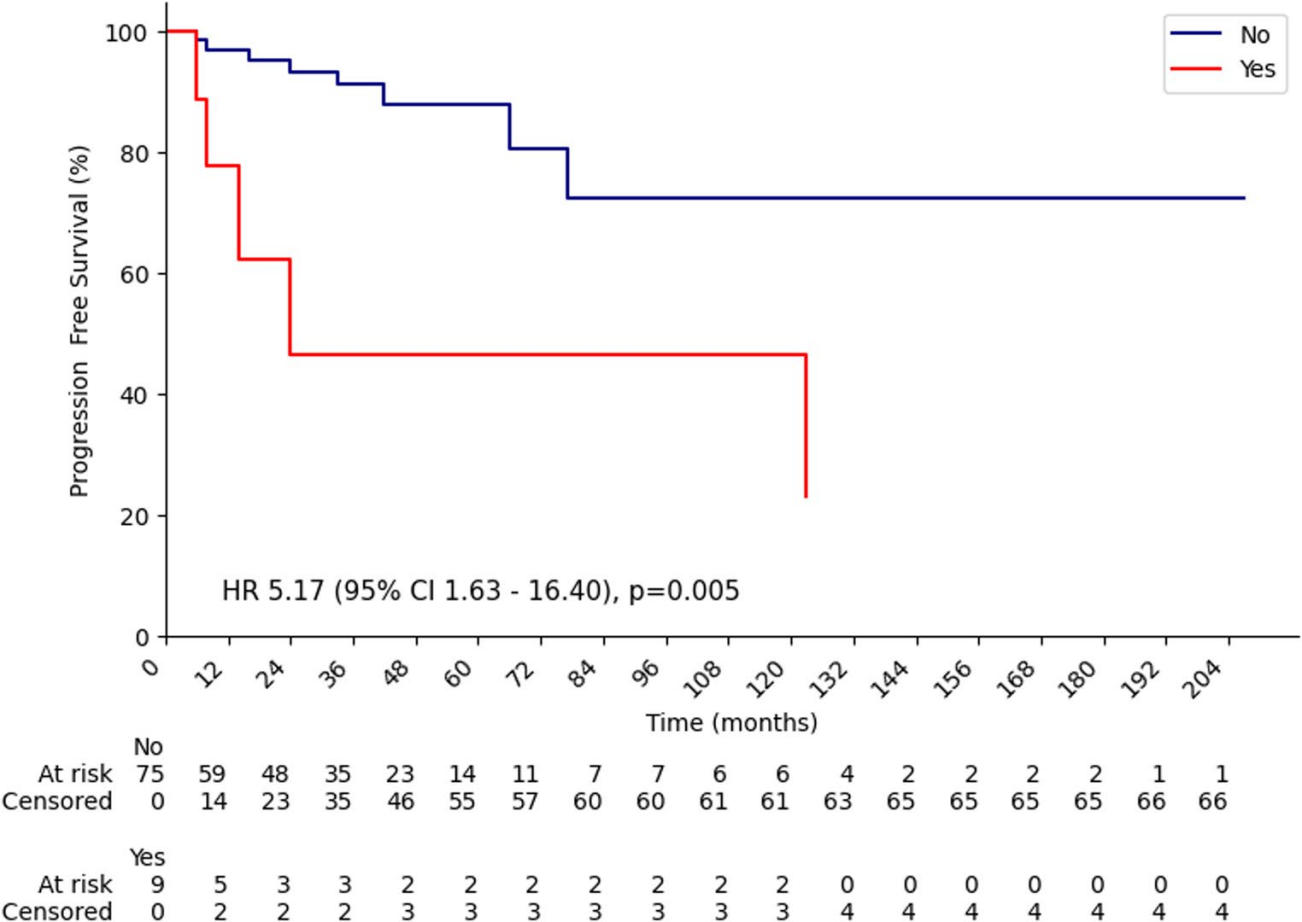
Kaplan–Meier progression-free survival (PFS) curves for the entire cohort, stratified by history of prior radiotherapy

In the overall cohort, the median PFS was 24 months in patients who had received prior radiotherapy (RT), whereas the median PFS was not reached in those without prior RT. The 1-year recurrence rates were 22.2% and 2.9%, and the 2-year recurrence rates were 53.3% and 6.6% in patients with and without prior RT, respectively (Fig. 2).

**Fig. 3:**
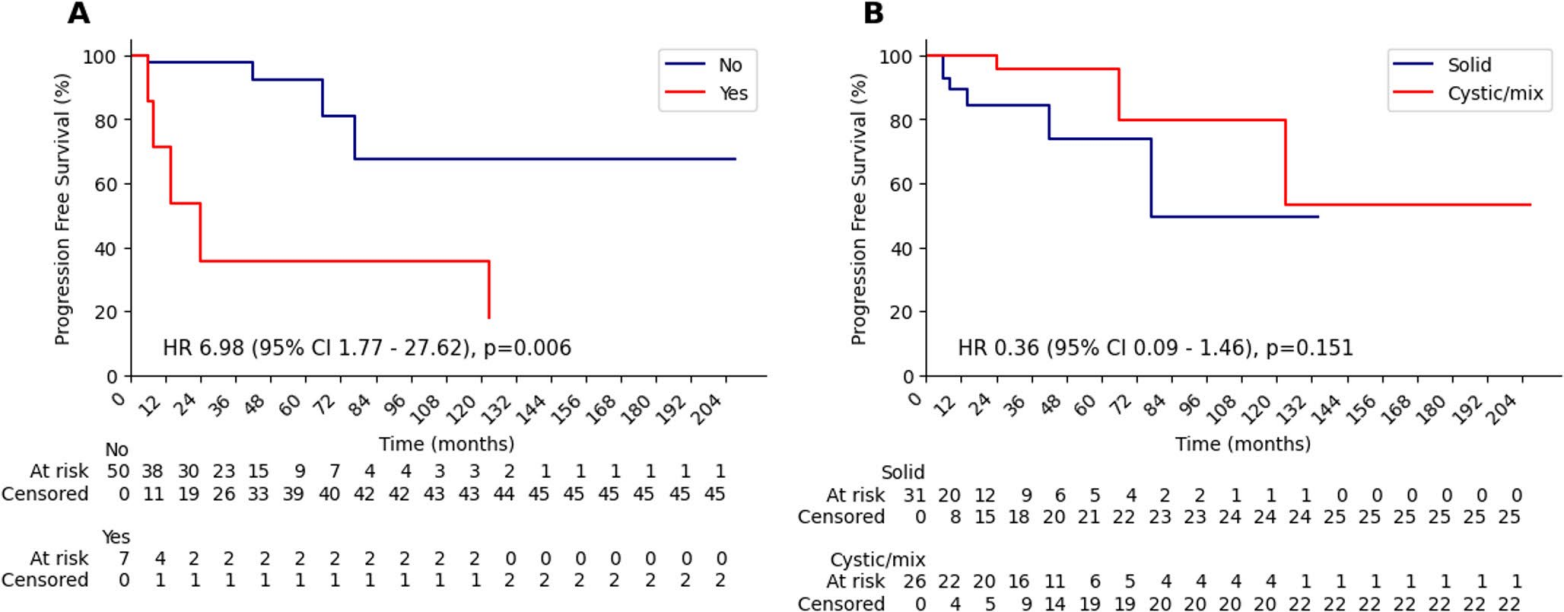
Kaplan–Meier progression-free survival (PFS) curves for the pediatric subgroup: (**A**) comparison based on prior radiotherapy status; (**B**) comparison according to tumor morphology (solid vs. cystic/mixed)

In the pediatric subgroup, the PFS curves stratified by prior RT status and by tumor type are presented in Fig. 3. Among patients who had received prior RT, the 1- and 2-year recurrence incidences were 28.6% and 64.3%, whereas in those without prior RT, the recurrence incidence during the first two years was 2.2%. For patients with cystic/mixed tumor morphology, the 1- and 2-year recurrence incidences were 11.1% and 15.5%, while in those with solid tumors, the recurrence incidence in the first two years was 4.5%.

Prophylactic corticosteroids were administered at the time of GKRS. All patients were discharged on the same day following treatment. No acute treatment-related complications were observed during the first three months of follow-up. No patients required surgical intervention or hospitalization for treatment-related adverse effects.

### Radiological outcomes

**Fig. 4:**
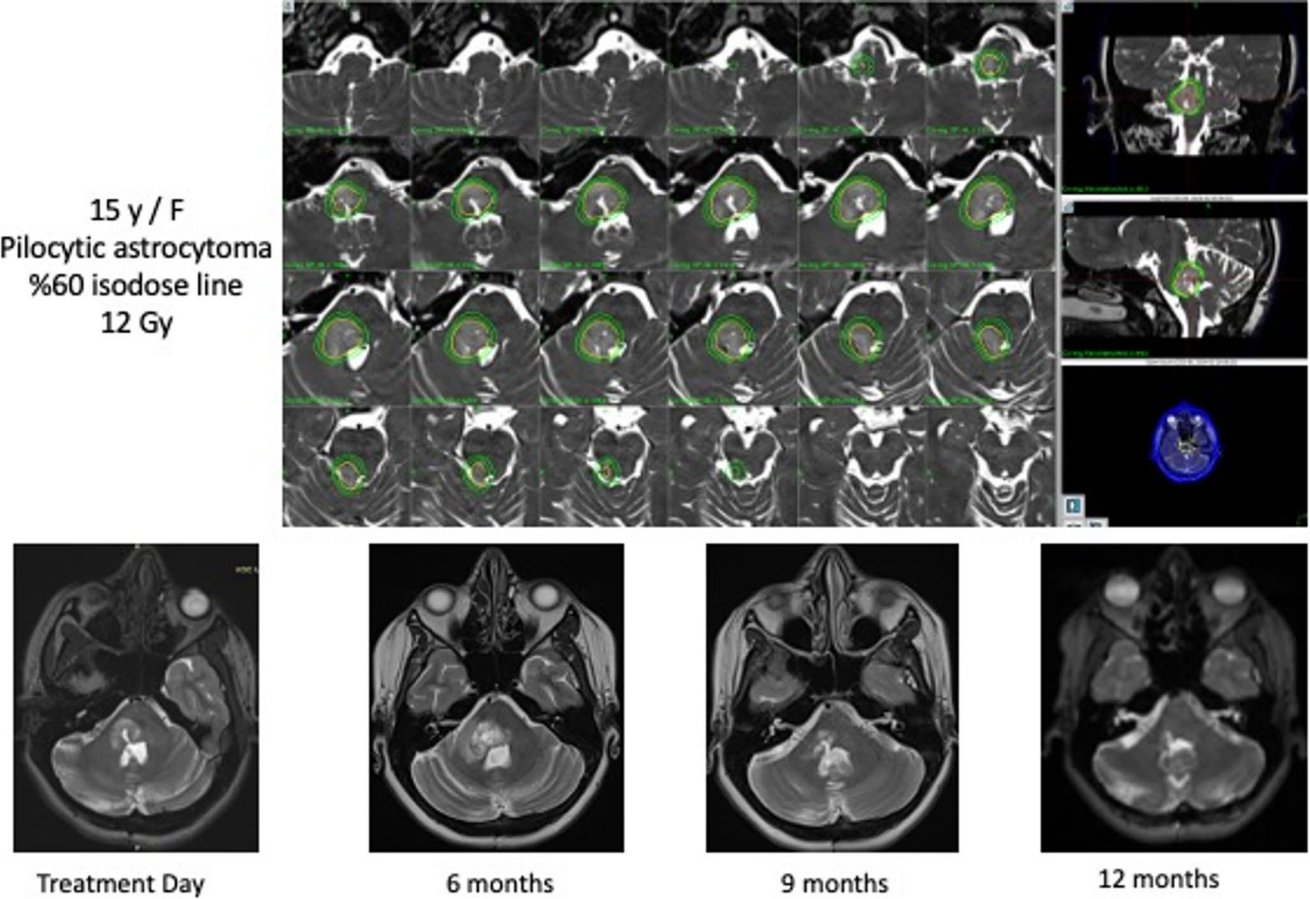
Gamma Knife radiosurgery dose planning and serial MRI follow-up of pilocytic astrocytoma

During median follow-up, 31 patients (36.9%) had no evidence of disease (CR) (Fig. 4), 15 patients (17.9%) showed partial response, 25 patients (29.8%) had stable disease, and 13 patients (15.5%) demonstrated progression in entire cohort. In the pediatric subgroup, 29 patients achieved CR, 8 had a partial response, 11 had stable disease, and 9 showed progression. In the adult group, 2 patients achieved CR, 7 had a partial response, 14 had stable disease, and 4 demonstrated disease progression.

These findings are consistent with the largest multicenter retrospective series published by Murphy et al. which analyzed 141 patients treated between 1990 and 2016, the 5- and 10-year PFS rates were reported as 74% and 69.7%, respectively [9]. The median age at GKRS in that cohort was 14 years. In the univariate analysis, age < 18 years, tumor volume < 4.5 cm³, and absence of prior RT or chemotherapy were identified as positive prognostic factors, whereas in the multivariate analysis, only prior RT remained a negative prognostic factor for PFS. Patients without prior radiotherapy demonstrated significantly better PFS rates at 1, 3, 5, and 10 years (92.3%, 85.7%, 78.8%, and 73.8%, respectively) compared to those who had undergone prior radiotherapy (76.2%, 54.4%, 48.4%, and 48.4%, respectively; p = 0.001). Similarly, in our study, prior RT was also identified as the only significant prognostic factor in the multivariate analysis. Among patients without a history of radiotherapy, the 1-, 3-, 5-, and 10-year PFS rates were 97.1%, 91.4%, 88%, and 72.6%, respectively, whereas in those who had received prior RT, the corresponding rates were 77.8%, 46.7%, 46.7%, and 23.3%. These findings are consistent with those reported in the largest multicenter series. It should be noted that the multicenter cohort reported by Murphy et al. likely includes patients from institutions previously represented in earlier single-center publications, including the cohorts described by Kano et al. Therefore, partial overlap between these datasets may exist. These studies are cited to provide historical and contextual comparison rather than to imply entirely independent patient populations.

Kano et al. reported outcomes of SRS for both pediatric and adult patients with pilocytic astrocytoma by analyzing these two cohorts separately [4, 8]. In their pediatric series of 50 patients who underwent SRS, the progression-free survival (PFS) rates for the entire cohort were 91.7%, 82.8%, and 70.8% at 1, 3, and 5 years, respectively [8]. In this study, univariate analysis demonstrated that newly diagnosed or residual tumor (p = 0.049), solid tumor morphology (p < 0.0001), tumor volume < 8 cc (p = 0.001), and absence of brainstem involvement (p = 0.009) were associated with improved PFS. Although prior RT (p = 0.175) did not show a significant effect on PFS, multivariate analysis results were not available. In comparison, in the pediatric subgroup of our series, the 1-, 3-, and 5-year PFS rates were 94.2%, 89.2%, and 84.7%, respectively. Furthermore, based on our multivariate analysis—which provided stronger statistical evidence—we found that absence of prior RT and solid tumor morphology were both significantly associated with improved PFS in pediatric patients.

Prior radiotherapy may represent an adverse prognostic factor for progression-free survival for several reasons. Patients who have previously received radiotherapy often have gross residual disease or recurrent tumors, indicating a more advanced disease course; consequently, Gamma Knife radiosurgery in these cases is frequently applied at a later stage, such as a second recurrence, which may be associated with inferior outcomes compared with treatment at first recurrence. In addition, prior irradiation may select for radioresistant tumor cell populations, further reducing the effectiveness of subsequent radiosurgical treatment. From a technical standpoint, when GKRS is delivered as a second course of radiation, prescription doses may be deliberately reduced to limit cumulative radiation toxicity, potentially compromising local tumor control.

In Kano et al.’s retrospective adult cohort, which included 14 patients, the 1-, 3-, and 5-year PFS rates were 83.9%, 31.5%, and 31.5%, respectively [4]. Although prior surgery was associated with improved PFS, no other significant prognostic factors were identified In our study, among 27 adults with available follow-up data, the 1-, 3-, and 5-year PFS rates were 96.4%, 81.7%, and 81.7%, respectively. In univariate analysis, neurological deficit, age, and brainstem location approached statistical significance; however, none remained significant in multivariate analysis. The adult subgroup analysis may be underpowered due to the limited number of patients and progression events, and these findings should therefore be interpreted with caution. Additionally, differences in baseline tumor characteristics may partly account for the observed variation in outcomes between studies. Notably, the proportion of patients with solid tumor morphology was 35.7% in the study by Kano et al., compared with 55.5% in our series. This difference in tumor morphology distribution may have contributed to differences in PFS; however, given the limited sample size, definitive conclusions cannot be drawn.

More recently, Wei et al. reported a 2023 retrospective analysis of 44 patients who underwent SRS for infratentorial pilocytic astrocytoma [11]. PFS rates were 95.4%, 79.0%, and 61.4% at 1, 5, and 10 years, respectively. In our study, 70.4% of patients had infratentorial tumors, and our corresponding PFS rates of 94.8%, 83.6%, and 59.3% were highly comparable to their findings, further supporting the effectiveness of GKRS in this subgroup.

Despite representing one of the largest single-center series in the literature, this study has several inherent limitations. The median follow-up duration of 36 months may not be sufficient to fully capture very late recurrences or delayed treatment-related effects, particularly in a predominantly pediatric and young adult population. Longer follow-up is therefore necessary to better define durable tumor control and long-term safety outcomes. The retrospective design limits the completeness and uniformity of long-term clinical and neurological outcome assessment. Although no acute treatment-related toxicity or symptomatic radionecrosis was observed, systematic long-term evaluation of neurocognitive and endocrine outcomes was not uniformly available in this cohort. Given the predominantly pediatric population, this represents an important limitation and highlights the need for prospective studies incorporating standardized functional outcome assessments. Given the exploratory nature of the subgroup analyses and the limited number of events, results should be interpreted cautiously, particularly in subgroup comparisons. Another limitation of this study is the lack of systematic molecular profiling according to current WHO classification standards, including BRAF fusion status. As many patients were treated before the routine incorporation of molecular diagnostics into standard practice, molecular subtype could not be evaluated as a prognostic or predictive factor. Future prospective studies integrating molecular characteristics with radiosurgical outcomes are warranted to better define biological predictors of treatment response. Nevertheless, the relatively large cohort size and the use of standardized survival methodology provide clinically meaningful data that contribute to the existing literature on Gamma Knife radiosurgery for pilocytic astrocytoma.

## Discussion

In this single-center study, which represents one of the largest institutional series of GKRS for pilocytic astrocytoma reported to date, the cumulative incidence of progression was 15.5% during a median follow-up of 36 months. In multivariate analysis, a history of prior radiotherapy (RT) was identified as an independent prognostic factor for treatment failure, while tumor morphology showed prognostic significance in the pediatric subgroup.

Previous Gamma Knife radiosurgery (GKRS) series in pilocytic astrocytoma have consistently demonstrated favorable progression-free survival (PFS), particularly in patients with relatively small tumor volumes. As summarized in Table 4, most published cohorts reported median tumor volumes ranging from approximately 2 to 5 cm³, with prescribed marginal doses typically between 13 and 15 Gy. In our study, the median treatment volume was 2.7 cm³, which is consistent with the range reported in the literature. Despite the use of a slightly lower median prescribed dose (12 Gy), long-term tumor control rates were comparable to previously published series. The majority of patients (51%) received a marginal dose of 12 Gy. Given the relatively narrow dose distribution and limited number of progression events, a formal dose–response analysis was not performed.

**Table 4:** Published Gamma Knife radiosurgery series for pilocytic astrocytoma and comparison with the current study

Author et al., Year	Cohort/Number	Treatment/Tumor Volume	Prescription Dose	PFS	Worse Prognostic Factors for PFS
Kano et al., 2009	Pediatric (Median age 10.5 years) *N*:50	Median tumor volume 2.1 cc (0.17–14.4 cc)	Median 14.5 Gy (11–22.5 Gy)	1-year:91.7% 3-year:82.8% 5-year: 70.8%	Recurrent lesion* Brainstem location * Cystic lesion *
Kano et al., 2009	Adult (Median age 32.3 years) *N*: 14	Median tumor volume 4.7 cc (0.6–33.7 cc)	Median 13.3 Gy (10–20 Gy)	1-year:83.9% 3-year:31.5% 5-year: 31.5%	No Surgery*
Murphy et al., 2021	Pediatric and Adult (Median age 14 years) N:141	Median tumor volume 3.45 cc (0.16, 33.70 cc)	Median 14 Gy (4–22.5 Gy)	1-year:89.9% 3-year:80.8% 5-year:74.0% 10-year:69.7%	Prior RT**
Wei et al., 2023	Pediatric and Adult (Median age 11.6 years) N:44	Median treatment volume 3.22 cc (0.16–26.60 cc)	Median 14 Gy (9.60–20 Gy)	1-year:95.4% 5-year:79.0% 10-year:61.4%	Margin Dose (< 14 Gy) **
Senyurek et al. (Current Study)	Pediatric and Adult (Median age 15 years) N: 88	Median treatment volume 2.7 cc (0.7–9.3 cc)	Median 12 Gy (8–15 Gy)	5-year:83.6% 10-year:71.2%	Cystic lesion** Prior RT**

*Abbreviations*: *PFS* Progression-free survival, *N* Number of patients, *Gy* Gray, * Univariate analysis, ** Multivariate analysis

As Murphy et al. represents a multicenter collaboration, partial patient overlap with previously published institutional series (e.g., Kano et al.) is possible

## Conclusion

Our study, representing one of the largest single-center series evaluating GKRS for pilocytic astrocytoma, demonstrates that GKRS provides durable long-term tumor control in both pediatric and adult patients. The strongest and most consistent predictor of recurrence across the cohort was a history of prior radiotherapy, which significantly reduced progression-free survival. In pediatric patients, cystic/mixed tumor morphology was also associated with a higher risk of recurrence, whereas no independent predictors were identified in the adult subgroup, likely reflecting sample size limitations.

Overall, our findings align with previously published multicenter and institutional series, reinforcing the effectiveness and safety of GKRS as a treatment modality for pilocytic astrocytoma. However, heterogeneity in clinical characteristics and the retrospective nature of the study underscore the need for prospective, standardized studies to better define optimal patient selection and long-term functional outcomes.

## Data Availability

No datasets were generated or analysed during the current study.
